# Supplementary material for: HEV ORF2 protein-antibody complex deposits are associated with glomerulonephritis in hepatitis E with reduced immune status
Source: Nat Commun. 2024 Oct 14;15:8849. doi: 10.1038/s41467-024-53072-0 (PMC11471813; doi:10.1038/s41467-024-53072-0)
Supplement: Supplementary file 1 — Supplementary Information [file 41467_2024_53072_MOESM1_ESM.pdf]

SUPPLEMENTARY INFORMATION

Anti-HEV antibody-ORF2 complex deposits are associated with glomerulonephritis in hepatitis E with reduced immune status

Running title: Hepatitis E-associated kidney disease

TABLE OF CONTENTS

SUPPLEMENTARY FIGURES
SUPPLEMENTARY TABLES
DETAILED CLINICAL INFORMATION, PATIENTS 1-4
Patient 1
Patient 2
Patient 3
Patient 4
DETAILED HISTOPATHOLOGIC DESCRIPTION, PATIENTS 1-4
Patient 1 – kidney specimens
Patient 1 – liver specimen
Patients 2-4 – kidney specimens
Patients 2-4 – liver specimens
REFERENCES

SUPPLEMENTARY FIGURES

Supplementary Figure 1

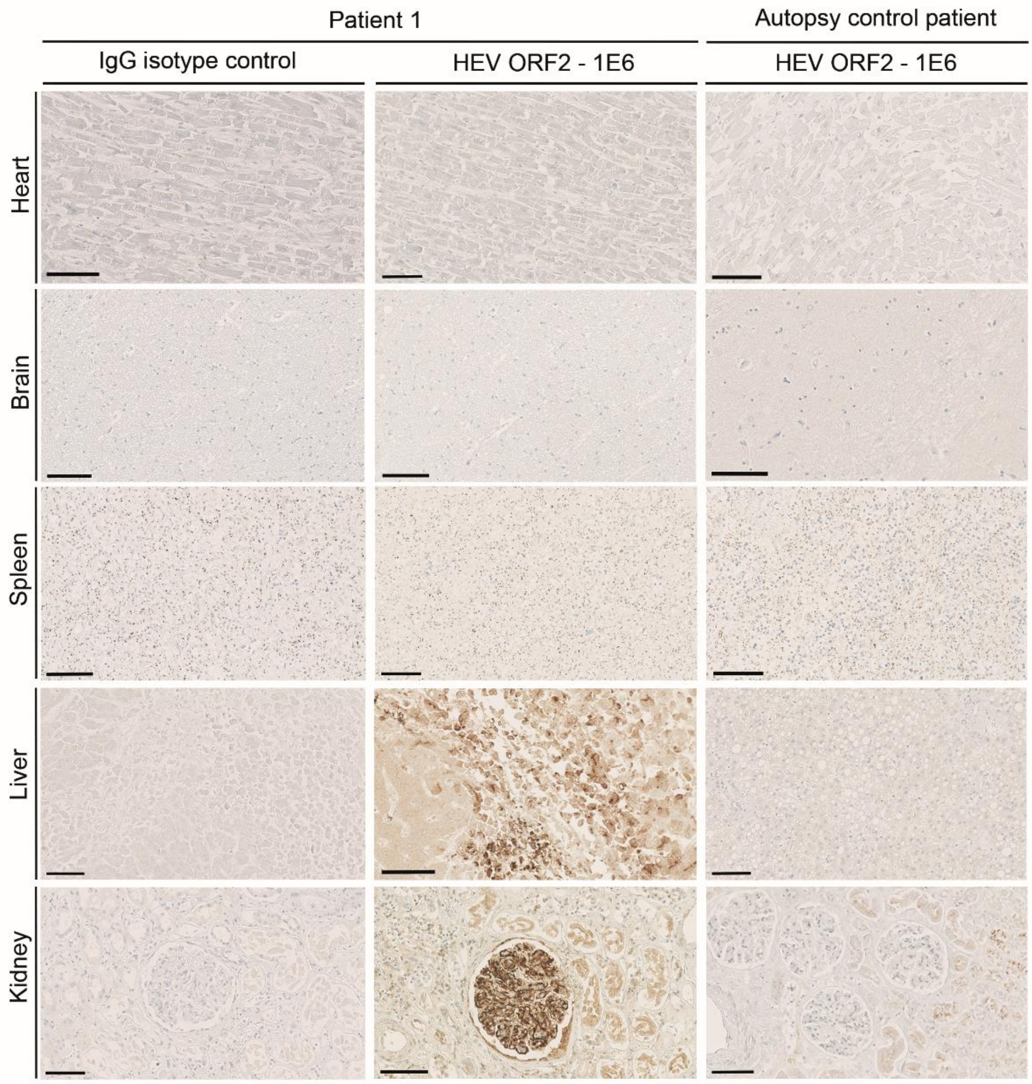

Supplementary Figure 1 HEV ORF2 immunohistochemistry in kidney transplant recipient with hepatitis E (Patient 1) and an autopsy control patient. Except in liver and kidney, no specific immunoreactivity for HEV ORF2 protein was obtained in heart, brain and spleen using 1E6 antibody. Corresponding areas. Scale bars: 50 µm.

Supplementary Figure 2

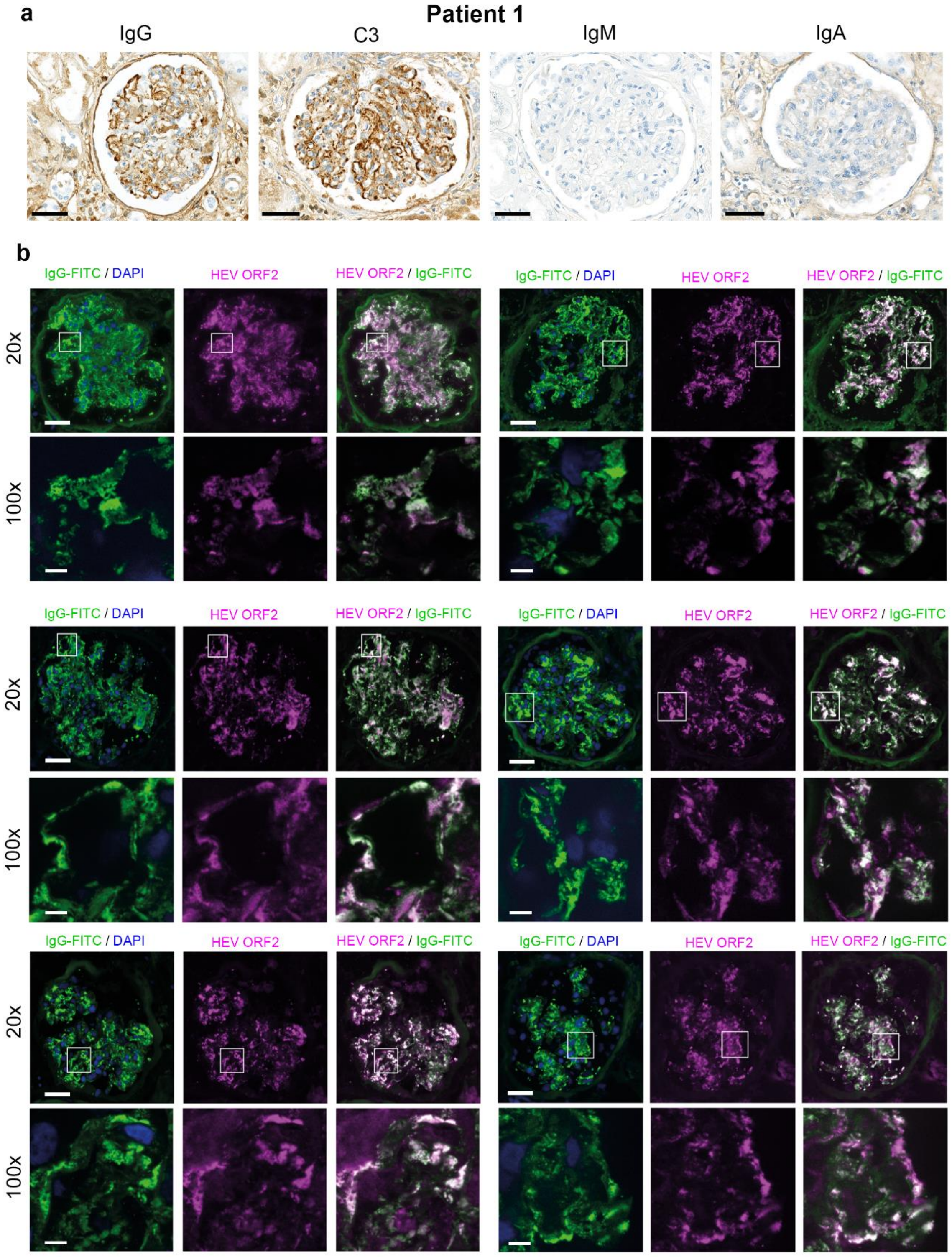

Supplementary Figure 2 Glomerular IgG/HEV ORF2 extracellular co-localization in a kidney transplant recipient with hepatitis E (Patient 1) (a) Immunohistochemistry for IgG with moderate (2+) mesangial and glomerular basement membrane deposits, C3 with moderate (2+) mesangial and glomerular basement membrane deposits, IgM and IgA with negative staining patterns. Scale bars: 50 µm. **(b)** Visualization by immunofluorescence

staining of six glomeruli from the autopsy transplant kidney (Patient 1) as in Figure 2A. IgG (left: green, FITC stain; DAPI counter-stain, blue) highlighting the co-localization with HEV ORF2 protein (middle: magenta, Alexa546 stain; right: overlay with white indicating co-localization). For each glomerulus, overview at low magnification (top rows, scale bar: 50 µm, 20x) and high-resolution images (bottom rows, scale bar: 5µm, 100x) corresponding to the areas indicated by the white boxes.

Supplementary Figure 3

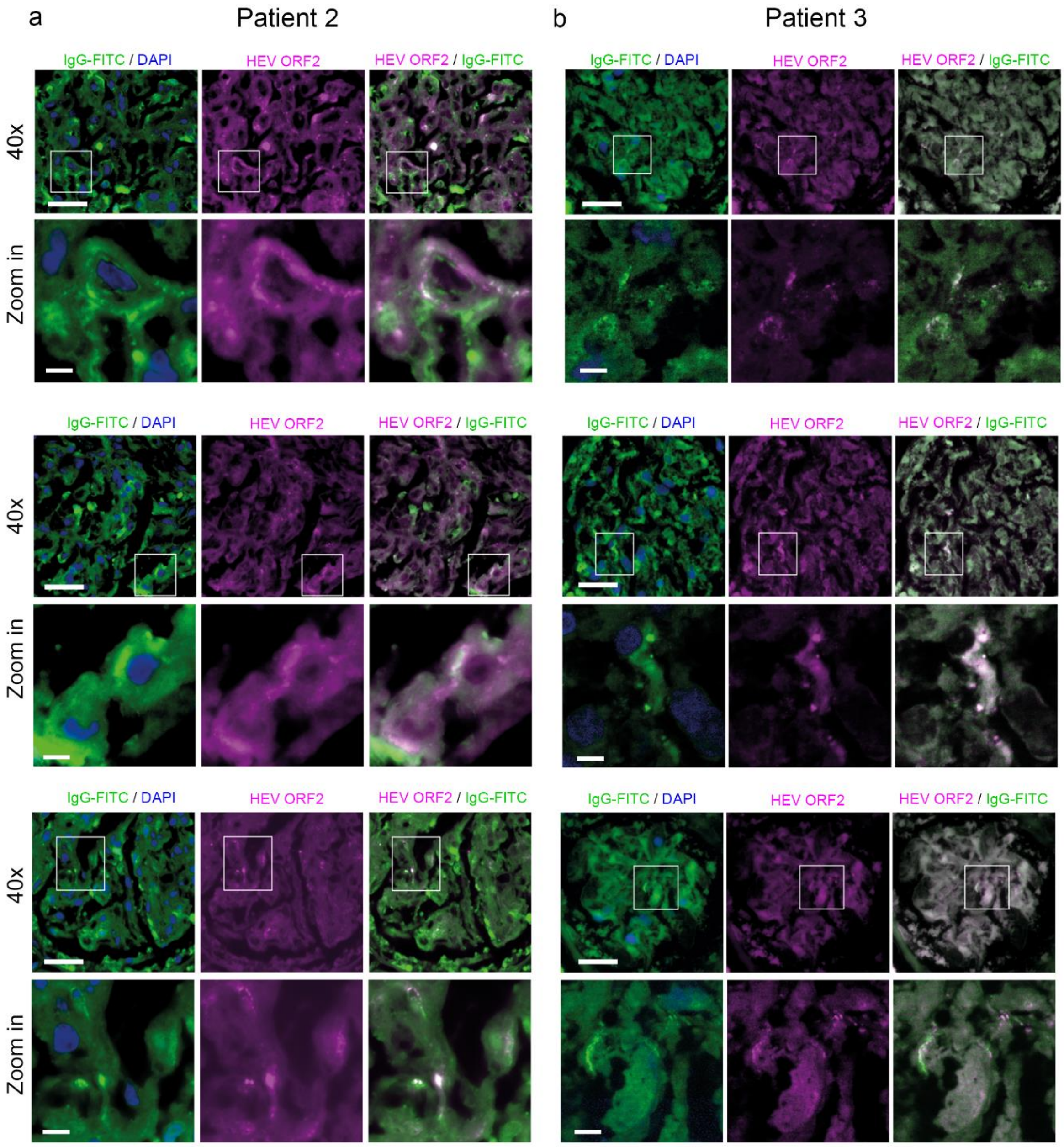

Supplementary Figure 3 Glomerular IgG/HEV ORF2 extracellular co-localization in patients with acute hepatitis E (a) Visualization by immunofluorescence staining of three glomeruli from patient 2, for which sparse, partially co-localized staining was found. IgG (left: green, FITC stain; DAPI counter-stain, blue) and HEV ORF2 protein (middle: magenta, Alexa546 stain; right: overlay with white indicating co-localization). For each glomerulus, overview at low magnification (top rows, scale bar: 30 µm, 40x) and zoom-in images (bottom rows, scale bar: 5 µm, zoom in) corresponding to the areas indicated by the white boxes. **(b)**

Same as in **(a)** for three glomeruli from patient 3. Here, high-resolution images (bottom rows) were obtained with the 100x objective and the ApoTome.

Supplementary Figure 4

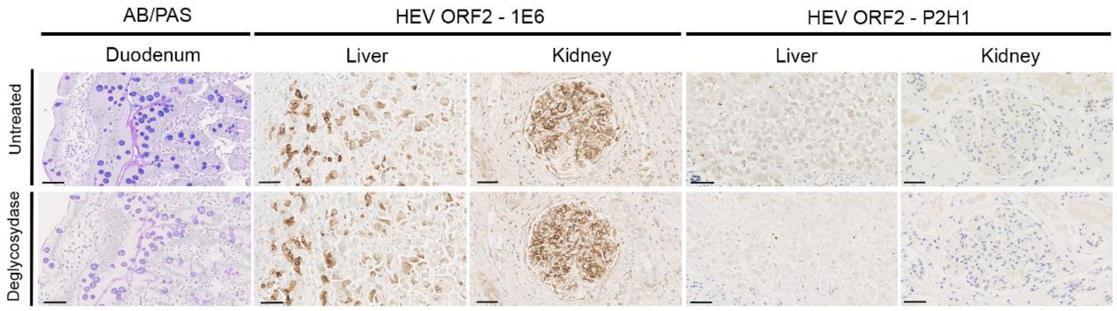

Supplementary Figure 4 On-slide deglycosylation of 1E6 and P2H1-stained liver and kidney tissue of Patient 1. AB/PAS-stained duodenum sections used as positive control, with a clear attenuation of the glycocalix staining. Corresponding areas. Scale bars: 50 µm.

SUPPLEMENTARY TABLES

Supplementary Table 1 Summary of initially and newly performed analyses and subsequent diagnostics for biopsies and autopsy materials of Patient 1.

	Patient 1 - kidney	Biopsy 1	Biopsy 2	Biopsy 3	Biopsy 4	Biopsy 5	Autopsy
		**1 month post-TPL**	**3.5 months post-TPL**	**6.5 months post-TPL**	**11 years and 3 months post-TPL**	**11 years and 4 months post-TPL**	**11 years and 7 months post-TPL**
	**Source**	University Basel Hospital, Switzerland			Cantonal Hospital Aarau, Switzerland		Zürich University Hospital, Switzerland
	**Material**	FFPE<br>FF			FFPE<br>FF<br>Glutaraldehyde fixed		
	**Histological description**	**Refer to supplementary materials, pages 6-11**					
**According to original reports**	**IHC**	(-) SV40	(+) SV40		Not mentioned		Not applicable
	**IF**	(-) IgA, IgM, C3<br>(-) C4d in peritubular capillaries	(-) IgG, IgA, IgM, and C3<br>(-) C4d in peritubular capillaries	(-) IgA, IgM, IgG,<br>(-) C4d in peritubular capillaries	(-) IgA, IgM, kappa and lambda, C4d<br><br>(+) IgG and C3, granular positivity in GBM and mesangium	(-) C4d, IgM<br>(+) glomerular IgG, kappa and lambda<br><br>IgA and C3 not evaluable	Not applicable
	**Diagnosis**	Mild focal tubular atrophy and interstitial fibrosis	Polyomavirus nephropathy	Polyomavirus nephropathy	Proliferative and focal sclerosing immune complex-mediated GN		Not applicable
**USZ workup**	**IHC**	(-) SV40	(+) SV40	(-) SV40*	(-) IgA and IgM, SV40<br>(+) IgG and C3 in GBM and mesangium	(-) IgA, C4d<br>(+) IgG, C3 in GBM and mesiangium	(-) SV40, IgA, IgM<br>(+) IgG, C3 in GBM and mesangium
	**HEV ORF2 IHC**	(-) HEV ORF2 (Figure 1c)			(+) HEV ORF2 (Figure 1c)		
	**IF**	Frozen tissue not available					(-) IgA, C4d<br>(+) IgG, kappa and lambda, C3
	**EM**	No electron dense deposit	Not done		Electron dense inhomogenously osmiophilic mesangial, subendothelial and subepithelial deposits. No substructure.		
	**Diagnosis**	Mild focal tubular atrophy and interstitial fibrosis.	Polyomavirus nephropathy.	Polyomavirus nephropathy. Mild arteriolosclerosis.	Proliferative and sclerosing immune complex-mediated GN consistent with HEV-associated GN		Proliferative and sclerosing immune complex-mediated GN with a membranoproliferative pattern, consistent with HEV-associated membranoproliferative GN with immune complexes
		No evidence for antibody-mediated rejection or recurrent IgA nephropathy					

Post-TPL, post-transplantation, USZ, University Hospital Zurich, FFPE: formalin-fixed, paraffin-embedded, FF: fresh frozen, GBM: glomerular basement membrane, EM: electron microscopy, GN: glomerulonephritis

*HE morphology was indicative of BK infection, suggestive for resolving BK infection.

Supplementary Table 2 Mass spectrometry results of liver, laser-captured interstitium and glomeruli.

	**Non-HEV-infected patient**			**Patient 1**		
**Total spectrum count**	**Liver**	**Interstitium**	**Glomeruli**	**Liver**	**Interstitium**	**Glomeruli**
HEV ORF2	**0**	0	0	**25**	4	**241 ± 47**
Podocin	n.d.	0	**17 ± 2**	n.d.	0	**13 ± 4**
Cytokeratin 7	n.d.	33	28	n.d.	20	29 ± 1
Complement 3	n.d.	74	114 ± 2	n.d.	23	236 ± 46
Collagen 1A1	70	12	22 ± 1	50	27	36 ± 2

Glomerular marker: podocin[1], renal markers: cytokeratin 7, complement 3 and collagen 1A1.[2] Results are expressed as total spectrum count with over 95% probability in mean ± s.d. n.d.: not detected.

DETAILED CLINICAL INFORMATION, PATIENTS 1-4

Patient 1

This male patient underwent renal transplantation at the age of 40 years for end-stage renal disease due to rapidly progressive crescentic IgA nephritis. Initial immunosuppression consisted of tacrolimus, mycophenolate mofetil (MMF) and sirolimus. Declining renal function prompted allograft biopsy four weeks post transplantation with focal tubulointerstitial fibrosis but no evidence of rejection or calcineurin inhibitor toxicity. His subsequent course was complicated by BK virus (BKV) nephropathy noted in a protocol biopsy 3.5 months post-transplant (post-TPL) and in a protocol biopsy 6.5 months post-TPL. Immunosuppression was reduced. Following BKV clearance and stabilization of renal function baseline, immunosuppression consisted of tacrolimus and MMF. Two years post transplantation the patient developed type 2 diabetes mellitus and was treated with oral antidiabetic agents.

Ten years after transplantation deranged liver function tests were noted (AST: 135 U/l, ALT: 156 U/L, 19 months prior to death). Hepatitis B surface (HBs) antigen and anti-hepatitis C virus were negative, anti-HBs and anti-hepatitis B core positive, consistent with previously known resolved hepatitis B. Drug history and history of alcohol consumption were inconspicuous. Further diagnostic work-up revealed liver cirrhosis of at the time unknown etiology, with splenomegaly, esophageal varices and ascites.

Eleven years and 3 months post-TPL (i.e. 4 months prior to death) rising creatinine levels, proteinuria and microhematuria triggered a renal transplant biopsy to rule out recurrent IgA nephropathy. A diagnosis of *de novo* immune complex glomerulonephritis (GN) positive for IgG and C3 on immunofluorescence was made and confirmed by the presence of subendothelial, mesangial and subepithelial electron dense deposits on electron microscopy. A focal interstitial inflammatory infiltrate and tubulitis were interpreted as being associated with the glomerulonephritis, although borderline changes suspicious for acute T-cell mediated rejection could not be excluded. When the patient was hospitalized for 8 days to adjust his immunosuppressive therapy, he presented with exertional dyspnea, night sweats and weight loss of 16 kg over the last six months upon admission. Steroid therapy was initiated. Given the

resolved hepatitis B virus infection, entecavir prophylaxis was initiated. Two weeks later the patient was readmitted for progressive renal failure and persistent nephritic syndrome. Repeat renal biopsy (3 months prior to death, 11 years and 4 months post-TPL) revealed persistent immune complex GN without evidence of rejection. Complement components C3/C4 were within normal limits. The patient was treated with intravenous (IV) pulses followed by oral steroids. Four weeks later he was admitted for renal allograft failure. Thrice-weekly hemodialysis was initiated. Transjugular liver biopsy was reported as chronic hepatitis with mild inflammatory activity on a background of bridging fibrosis or cirrhosis.

Two weeks before his death the patient was readmitted for further evaluation of deteriorating liver function. He had noted increased jaundice and pruritus. He also reported dyspnea and productive cough. Intravenous albumin for severe hypoalbuminemia and vitamin K substitution were initiated. Serologic tests were positive for anti-HEV IgG and negative for anti-HEV IgM (VIDAS® ANTI-HEV IgG and IgM assays, BioMérieux, France). Eight days before death the patient was transferred to a tertiary care center for evaluation of liver transplantation. The following tests were negative / inconspicuous: α1-antitrypsin, anti-double stranded DNA antibodies, rheumatoid factor, ANCA, MPO-ANCA, PR3-ANCA, anti-glomerular basement membrane antibodies, hepatitis A IgM antibodies, hepatitis B surface antigen, hepatitis B core IgM antibody, hepatitis B e-antigen antibodies, hepatitis D virus antibodies, hepatitis C virus antibodies, HIV-1 and HIV-2 antigen and antibody screen, human T-cell lymphotropic virus types I and II antibody, PCR for HBV DNA, HCV RNA, CMV DNA and BKV DNA. Tests for hepatitis A IgG antibodies, hepatitis B surface antibodies, hepatitis B core antibodies, hepatitis E virus IgG antibodies were positive. In contrast to previous testing, also hepatitis E virus IgM antibodies were now detectable (Enzyme ImmunoAssays provided by Dia. Pro Diagnostic Bioprobes Srl, Italy) probably due to the different serological tests used.[3] Over the next days, the patient became hemodynamically unstable and developed hepatic encephalopathy. A chest CT scan showed bilateral infiltrates consistent with nosocomial versus aspiration pneumonia. Despite maximal intensive care treatment, he developed multi-organ dysfunction syndrome. In the absence of therapeutic options, supportive care was initiated. The patient

died the following day, 11 years and 7 months after kidney transplantation. The HEV RNA PCR result, which became available only after the patient's death, was positive with very high viremia of 1.2×10^{8} IU/mL.

Patient 2

This 59-year-old male patient with known liver cirrhosis due to nonalcoholic steatohepatitis, metabolic syndrome and history of coronary artery disease, was admitted to a regional hospital because of acute-on-chronic liver disease. Initial laboratory tests revealed elevated serum aminotransferases and cholestatic parameters, hyperbilirubinemia, decreased albumin and fibrinogen levels as well as prolonged prothrombin time. Further analysis revealed positive anti-HEV IgG and IgM antibodies and HEV RNA of 4.0×10^{6} IU/mL, consistent with acute hepatitis E. Transient deterioration of renal function (maximum creatinine level 150 µmol/L, minimal GFR 42 mL/min/1.73 m^2) was interpreted as stage1 acute kidney injury. Renal function improved after treatment with IV albumin. After initial improvement of hepatic and renal parameters, the clinical course was complicated by an upper gastrointestinal bleeding, which led to an acute deterioration of hepatic and renal function (max. creatinine 188 µmol/L, minimal eGFR 33 mL/min/1.73 m^2). The patient developed hepatic encephalopathy and was transferred to a tertiary care center. At admission, the patient was anuric and required hemodialysis which was attributed to hepato-renal syndrome. A transjugular liver biopsy confirmed the clinically suspected cirrhosis. Three days after admission, the patient experienced severe hemodynamic instability due to recurrent bleeding from duodenal ulcerations. Four days after admission to the tertiary care center, the patient developed severe distributive and septic shock with marked coagulopathy and died. Autopsy confirmed acute-on-chronic liver disease and duodenal ulcerations.

Patient 3

This 66-year-old female patient with a history of type 2 diabetes mellitus (treated with oral antidiabetic agents) was admitted to a Cantonal Hospital for suspected acute-on-chronic liver

disease. A CT scan revealed liver cirrhosis with mild splenomegaly and moderate ascites in all four quadrants. Laboratory tests revealed elevated levels of serum aminotransferases, cholestatic parameters, hyperbilirubinemia, increased INR and elevated ammonia levels. The hemogram was normal, including a normal platelet count. There was no evidence of hepato-renal syndrome with only slightly elevated urea (9.9 mmol/L), but otherwise normal renal retention parameters (creatinine 70 µmol/L, eGFR 95 mL/min/1.73 m^2). Hepatopathy screening was positive for HEV RNA (4.6 x 10^4 IU/mL) as well as HEV IgG and IgM antibodies, consistent with acute hepatitis E on top of pre-existing liver disease. Antiviral therapy with ribavirin was initiated. A transjugular liver biopsy confirmed cirrhosis. During hospitalization, the patients renal function deteriorated considerably from day 9 after admission, which was interpreted as hepato-renal syndrome (values two weeks after admission: creatinine 180 µmol/L, eGFR 25 mL/min/1.73 m^2, urea 25 mmol/L). The patient subsequently developed hepatic encephalopathy and was transferred to a tertiary care center. Within hours after admission, rapid neurological and respiratory deteriorations were noted. After anuric kidney failure, progressive encephalopathy and respiratory failure with pulmonary edema, the patient experienced severe distributive shock with marked coagulopathy and succumbed one day later. Autopsy confirmed acute-on-chronic liver disease.

Patient 4

This 76-year-old male patient was admitted to a tertiary care center for suspected acute-on-chronic liver disease. He had a history of alcoholic liver cirrhosis, metabolic syndrome with obesity, insulin resistance and arterial hypertension. Laboratory tests revealed elevated serum aminotransferases and cholestatic parameters, hyperbilirubinemia and hypoalbuminemia (24 g/L). A transjugular liver biopsy confirmed liver cirrhosis. Further laboratory tests revealed positive anti-HEV IgG and IgM antibodies as well as HEV RNA of 2.2 x 10^3 IU/mL, consistent with acute hepatitis E. Antiviral therapy with ribavirin was initiated. Ribavirin treatment was discontinued five days later due to worsening renal function. From day 9 after admission hepatic encephalopathy as well as the renal function worsened, the latter diagnosed as hepato-

renal syndrome. From day 11 after admission, the renal retention parameters worsened significantly (creatinine > 200 µmol/L), prompting therapy with terlipressin and albumin. However, there was no improvement, requiring initiation of hemodialysis. In view of the dramatic deterioration, treatment was switched to comfort care, and the patient died 16 days after admission to the tertiary care center. An autopsy was performed which confirmed the acute-on-chronic liver disease.

Clinical findings obtained in patients 2-4 with respect to their liver phenotype were recently described in Vieira Barbosa et al.[4]

DETAILED HISTOPATHOLOGIC DESCRIPTION, PATIENTS 1-4

Patient 1 – kidney specimens

Staining, electron microscopy analysis and diagnostics are summarized in supplementary table 1.

Allograft kidney biopsy (1 month post-transplantation (post-TPL), Figure 1c):

Light microscopy revealed a core of renal cortex containing up 12 glomeruli all with unremarkable features on light microscopy. Minimal interstitial fibrosis and tubular atrophy comprising <5% of the cortex, few assessable arterioles and arteries with unremarkable features were found. No peritubular capillaritis was found.

Immunofluorescence (as stated in the original biopsy report) revealed negative staining results for IgA, IgM and C3 in the glomeruli. IgG was not evaluable. C4d was negative in peritubular capillaries.

Immunohistochemistry (as stated in the original biopsy report): SV40 was negative.

Immunohistochemistry (performed for this study): HEV ORF2 protein: Negative.

Electron microscopy was not performed for initial evaluation.

Electron microscopy (performed for this study) on renal tissue obtained from the paraffin block revealed no electron dense deposits.

Diagnosis: Mild focal tubular atrophy and interstitial fibrosis. No evidence of rejection, polyomavirus nephropathy or recurrent IgA nephropathy.

Allograft kidney biopsy (3.5 months post-TPL):

Light microscopy: Renal cortex with 18 glomeruli, 2 hyalinized. Focal interstitial edema and predominantly lymphocytic infiltrates comprising approximately 10-20% of the cortex, with tubulitis and intranuclear inclusions in the tubular epithelium typical for polyomavirus. Focal interstitial fibrosis and tubular atrophy, involving approximately 5-10% of the cortex. One artery with mild intimal fibrosis and elastosis. Some arteries and arterioles with swollen myocytes with pale cytoplasm.

Immunofluorescence (as stated in the biopsy report): IgA, IgG, IgM and C3 negative in glomeruli. C4d negative in peritubular capillaries.

Immunohistochemistry (as stated in the original biopsy report): SV40 focally positive in nuclei of tubular epithelial cells.

Immunohistochemistry (performed for this study): HEV ORF2 protein: Negative.

Electron microscopy was not performed.

Diagnosis: Polyomavirus nephropathy. Minimal interstitial fibrosis and tubular atrophy. Minimal signs of calcineurin-inhibitor-associated toxicity on praeglomerular vessels. No evidence of antibody-mediated rejection. No evidence of recurrent IgA nephropathy.

Allograft kidney biopsy (6.5 months post-TPL):

Light microscopy: Cortex with 7 glomeruli, two with fibrosis of the capsule. Focal interstitial fibrosis and tubular atrophy with lymphocytic and plasma cell infiltrates. Focal edema and infiltration of lymphocytes, macrophages and many plasma cells. Tubulitis with lymphocytes and plasma cells in tubules with intranuclear inclusions in tubular epithelium typical for polyomavirus. Two arteries with segmental sclerosis of the wall. Mild arteriolosclerosis.

Immunofluorescence (as stated in the original biopsy report): IgA, IgG, IgM and C3 negative in glomeruli. C4d negative in peritubular capillaries.

Immunohistochemistry (as stated in the original biopsy report): SV40 focally positive in nuclei of tubular epithelial cells.

Immunohistochemistry (performed for this study): HEV ORF2 protein: Negative. SV40: Negative (despite morphology suggestive of polyomavirus infection and initial positive results outside).

Electron microscopy was not performed.

Diagnosis: Polyomavirus nephropathy. Mild arteriolosclerosis. No evidence of antibody-mediated rejection. No evidence of recurrent IgA nephropathy.

Allograft kidney biopsy (4 months prior to death, Figure 1c):

Light microscopy revealed 2 cores of cortex and medulla containing up to 8 glomeruli, two of which were hyalinized. Two glomeruli showed segmental sclerosis, one of those also endocapillary hypercellularity and prominent podocytes. Two glomeruli had mild mesangial expansion with minimal hypercellularity and mild increase in endocapillary mononuclear cells and neutrophils. Trichrome stain revealed diffuse, chunky mesangial and few glomerular basement membrane deposits, some suspicious for subepithelial “humps”.

The glomerular basement membrane showed rare holes and very rare splitting. Further findings included minimal interstitial fibrosis and tubular atrophy comprising <5% of the cortex, focal plasma cell-rich interstitial infiltrate involving less than 25% of the unscarred cortical parenchyma and moderate tubulitis with up to 10 leukocytes per tubular cross section, moderate arteriolar hyalinosis, and arteries with mild fibrointimal thickening. No peritubular capillaritis was found.

Immunofluorescence (as stated in the original biopsy report) revealed moderate (2+) granular mesangial and glomerular basement membrane positivity of IgG and C3. IgA, IgM, kappa and lambda were negative. C4d negative in peritubular capillaries.

Immunohistochemistry (performed for this study): Granular mesangial and glomerular basement membrane positivity for IgG and C3 (1+). IgA and IgM were negative. SV40: Negative.

HEV ORF2 protein: Mild granular positivity in mesangium and glomerular basement membrane (1+).

Electron microscopy showed expanded mesangium with increased matrix and electron dense deposits. The lamina densa was irregularly thickened. There were subepithelial electron dense deposits, some of them hump-like, intramembranous and subendothelial deposits. Deposits were inhomogenously osmiophilic, but without substructure. There were no deposits in the tubular basement membranes.

Primary diagnosis: Proliferative and focal sclerosing immune complex-mediated glomerulonephritis. Focal interstitial infiltrates and tubulitis, consistent with accompanying inflammation. No evidence of IgA nephropathy.

Diagnosis (upon re-evaluation and additional staining): Proliferative and focal sclerosing immune complex-mediated glomerulonephritis with positivity for HEV ORF2 protein, consistent with HEV-associated glomerulonephritis. No evidence of IgA nephropathy, antibody-mediated rejection or polyomavirus nephropathy.

Allograft kidney biopsy (3 months prior to death, Figure 1c)

Light microscopy revealed a core of renal cortex containing up to 9 glomeruli, 4 of which were hyalinized, 2 showed segmental sclerosis. The mesangium was mildly expanded, more than in the previous biopsy, with minimal, focal and segmental hypercellularity. There was very mild focal and segmental endocapillary hypercellularity with mononuclear cells and neutrophils. Trichrome stain revealed chunky mesangial and more glomerular basement membrane deposits, some suspicious for subepithelial “humps”. The glomerular basement membrane showed rare holes and rare splitting. Minimal interstitial fibrosis and tubular atrophy comprising <5% of the cortex were found, focal severe arteriolar hyalinosis and arteries with mild fibrointimal thickening, but no peritubular capillaritis.

Immunofluorescence (as stated in the original biopsy report) revealed mild glomerular peripheral basement membrane and mesangial positivity for IgG (1+). IgM was negative.

Slides stained for IgA and C3 did not show any glomeruli.

Kappa and lambda light chains were positive.

Immunohistochemistry (performed for this study): Granular mesangial and glomerular basement membrane positivity for IgG (1+ to 2+) and C3 (1+). IgA was negative. IgM was not available. C4d was negative in peritubular capillaries.

HEV ORF2 protein: Moderate granular to chunky positivity in mesangium and glomerular basement membrane (2+).

Electron microscopy was similar to the findings in the previous biopsy, but revealed even more deposits. Reticular aggregates were found in the cytoplasm of one endothelial cell.

Primary diagnosis: Proliferative and focal sclerosing immune complex-mediated glomerulonephritis suggestive for viral infections.

Diagnosis (upon re-evaluation and additional stainings): Proliferative and focal sclerosing immune complex-mediated glomerulonephritis with positivity for HEV ORF2 protein**, consistent with HEV-associated glomerulonephritis. No evidence of IgA nephropathy, rejection or polyomavirus nephropathy.**

Autopsy material of allograft kidney (11 years and 7 months after kidney transplantation, Figure 1c)

Light microscopy showed more mesangial expansion and mild hypercellularity, still in a focal and segmental pattern. There was more endocapillary proliferation with mononuclear cells and some neutrophils, more splitting of the glomerular basement membranes. Trichrome stain revealed irregular deposits in the mesangium and glomerular basement membrane, some suspicious for subepithelial “humps”. Some glomeruli showed segmental sclerosis, some were hyalinized. There was focal interstitial fibrosis and tubular atrophy comprising approximately 20% of the cortex, moderate arteriolar hyalinosis not involving smooth muscle cells and moderate fibrointimal thickening in arteries, but no peritubular capillaritis.

Immunofluorescence (performed for this study) showed moderate to strong (2-3+) mesangial and glomerular basement membrane deposition of IgG, moderate (2+) mesangial and glomerular basement membrane deposition of C3, weak to moderate (1-2+) mesangial and glomerular basement membrane deposition of kappa light chains, moderate (2+) mesangial and glomerular basement membrane deposition of lambda light chains. Traces of IgM were detected in the mesangium and glomerular basement membrane. IgA was negative.

C4d was negative in peritubular capillaries.

For immunofluorescent double staining IgG/HEV ORF2 protein, please refer to main manuscript, results section.

Immunohistochemistry (performed for this study) revealed moderate (2+) mesangial and glomerular basement membrane deposits of IgG and C3. IgA and IgM were negative. SV40 was negative.

HEV ORF2 protein: Strong granular to chunky positivity in mesangium and glomerular basement membrane (3+).

Electron microscopy (performed for this study) was similar to the findings in the biopsies.

Diagnosis: Proliferative and focal sclerosing immune complex-mediated glomerulonephritis with a membranoproliferative pattern and positivity for HEV ORF2 protein**, consistent with HEV-associated membranoproliferative glomerulonephritis with immune complexes. No evidence of IgA nephropathy. No evidence of rejection. No evidence of polyomavirus nephropathy.**

Patient 1 – liver specimen

Autopsy liver (shown in Figure 1b):

Light microscopic examination showed cirrhotic liver parenchyma with mild chronic active hepatitis, severe predominantly canalicular bile stasis and advanced autolytic changes. Immunohistochemistry revealed patchy areas of hepatocytes positive for HEV ORF2 protein, mostly showing a cytoplasmic, but also a nuclear staining pattern.[5]

Diagnostic: Chronic hepatitis E

Patients 2-4 – kidney specimens

Patient 2

Autopsy kidney (shown in Table 1):

Histologic findings in kidney tissue obtained from patient 2 showed glomeruli with minimal endocapillary hypercellularity with mononuclear cells and pigmented tubular cast. Immunohistochemistry revealed moderate mesangial positivity for HEV ORF2 protein (2+), IgG (1+ to 2+), IgM (2+) and trace IgA and C3. Collectively, these findings were consistent with hepatitis E-associated proliferative immune complex GN and bile cast nephropathy. For immunofluorescent double staining IgG/HEV ORF2 protein, please refer to main manuscript, results section.

Patient 3

Autopsy kidney (shown in Table 1):

Histologic findings in kidney tissue obtained from patient 3 showed autolytic changes with rare preserved cells in glomeruli and no preserved cells in tubules. The mesangium was expanded, consistent with diabetic glomerulosclerosis. Immunohistochemistry revealed moderate mesangial positivity for HEV ORF2 protein (2+), mild for IgG (1+), moderate IgM (2+), trace IgA and no C3. Collectively, these findings were consistent with hepatitis E-associated immune complex deposits without overt GN. In addition, some pigmented tubular casts were found, consistent with bile cast nephropathy. For immunofluorescent double staining IgG/HEV ORF2 protein, please refer to main manuscript, results section.

Patient 4

Autopsy kidney (shown in Table 1):

Histologic findings in kidney tissue obtained from patient 4 showed rare preserved cells in glomeruli and no preserved cells in tubules. Inconspicuous glomeruli with autolytic changes. Immunohistochemistry revealed mild mesangial positivity for HEV ORF2 protein (1+) and trace IgM. IgG, IgA and C3 were negative suspicious for very mild hepatitis E-associated deposits without overt GN. In addition, some pigmented tubular casts were found, consistent with bile cast nephropathy. Material not suitable for EM.

Patients 2-4 – liver specimens

Histologic findings obtained in livers of patients 2 – 4 were recently described in Lenggenhager et al.[6], with patient 2 of this study corresponding to patient 35, patient 3 of this study corresponding to patient 27, and patient 4 of this study corresponding to patient 31.

REFERENCES

[1] Boute N, Gribouval O, Roselli S, et al. NPHS2, encoding the glomerular protein podocin, is mutated in autosomal recessive steroid-resistant nephrotic syndrome. *Nat Genet* **24**, 349-54 (2000). DOI: 10.1038/74166.

[2] Barwinska D, El-Achkar TM, Melo Ferreira R, et al. Molecular characterization of the human kidney interstitium in health and disease. *Sci Adv* **7**, (2021). DOI: 10.1126/sciadv.abd3359.

[3] Kamar N, Izopet J, Pavio N, et al. Hepatitis E virus infection. *Nat Rev Dis Primers* **3**, 17086 (2017). DOI: 10.1038/nrdp.2017.86.

[4] Vieira Barbosa J, Mullhaupt B, Brunner F, et al. Autochthonous hepatitis E as a cause of acute-on-chronic liver failure and death: histopathology can be misleading but transaminases may provide a clue. *Swiss Med Wkly* **151**, w20502 (2021). DOI: 10.4414/smw.2021.20502.

[5] Lenggenhager D, Gouttenoire J, Malehmir M, et al. Visualization of hepatitis E virus RNA and proteins in the human liver. *J Hepatol* **67**, 471-9 (2017). DOI: 10.1016/j.jhep.2017.04.002.

[6] Lenggenhager D, Pawel S, Honcharova-Biletska H, et al. The histologic presentation of hepatitis E reflects patients' immune status and pre-existing liver condition. *Mod Pathol* **34**, 233-48 (2021). DOI: 10.1038/s41379-020-0593-1.
